# Computational investigation of the Mg-ion conductivity and phase stability of MgZr_4_(PO_4_)_6_†

**DOI:** 10.1039/c9ra00513g

**Published:** 2019-04-23

**Authors:** Koki Nakano, Yusuke Noda, Naoto Tanibata, Masanobu Nakayama, Koichi Kajihara, Kiyoshi Kanamura

**Affiliations:** Department of Advanced Ceramics, Nagoya Institute of Technology Gokiso, Showa Nagoya Aichi 466-8555 Japan masanobu@nitech.ac.jp; Center for Materials Research by Information Integration (CMI2), Research and Services Division of Materials Data and Integrated System (MaDIS), National Institute for Materials Science (NIMS) 1-2-1 Sengen Tsukuba Ibaraki 305-0047 Japan; Elements Strategy Initiative for Catalysts and Batteries (ESICB), Kyoto University 1-30 Goryo-Ohara, Nishikyo Kyoto 615-8245 Japan; Frontier Research Institute for Materials Science (FRIMS), Nagoya Institute of Technology Gokiso, Showa Nagoya Aichi 466-8555 Japan; Global Research Center for Environment and Energy based on Nanomaterials Science (GREEN), National Institute for Materials Science (NIMS) 1-1 Namiki Tsukuba Ibaraki 305-0047 Japan; Department of Applied Chemistry for Environment, Graduate School of Urban Environmental Sciences, Tokyo Metropolitan University 1-1 Minami-Osawa, Hachioji Tokyo 192-0397 Japan

## Abstract

Solid electrolyte materials exhibiting high Mg-ion conductivity are required to develop Mg-ion batteries. In this study, we focused on a Mg-ion-conducting solid phosphate based electrolyte, MgZr_4_(PO_4_)_6_ (MZP), and evaluated the ionic conductivity of NASICON-type and β-iron sulfate-type MgZr_4_(PO_4_)_6_ structures *via* density functional theory calculations. The calculations suggest that the migration energy of Mg is 0.63 eV for the NASICON-type structure and 0.71 eV for the β-iron sulfate-type one, and the NASICON-type structure has higher ion conductivity. Although the NASICON-type MZP structure has not been experimentally realised, there is only an energy difference of 14 meV per atom with respect to that of the β-iron sulfate-type structure. Therefore, in order to develop a synthesis method for the NASICON-type structure, we investigated pressure- and temperature-dependent variations in the free energy of formation using density functional perturbation theory calculations. The results suggest that the formation of the NASICON-type structure is disfavoured under the 0–2000 K and 0–20 GPa conditions.

## Abstract 2

Computational studies on Mg ion conduction in the MgZr_2_(PO_4_)_6_ with both NASICON-type and β sulfate-type structures have been performed.

## Graphical abstract

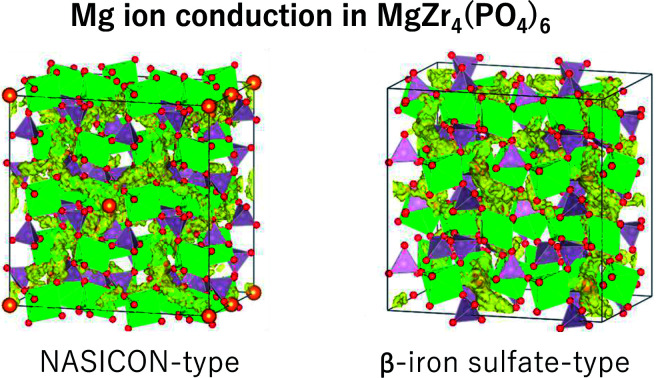

## Introduction

Lithium ion batteries (LIBs) are widely used in portable electronic devices, such as mobile phones and laptop computers, owing to their high energy density and long cycle life. In recent years, the use of (hybrid) electric vehicles (EVs) has gradually increased to address the environmental and energy issues.^1^ Therefore, LIBs are also attracting attention as secondary batteries for on-vehicle use. However, the following two major technical drawbacks of current LIBs need to be overcome: (i) insufficient driving range of the vehicle compared to the conventional petro-powered vehicle due to the small energy density and (ii) safety concerns arising from inflammable organic electrolytes. In this respect, developing all solid-state Mg-ion batteries (MIBs) is believed to be one of the ultimate solutions to resolve the above two issues.^2^ The Mg ion is divalent and can carry two electrons, so that the energy density is expected to double. All solid-state battery technology also contributes to the fabrication of a bipolar-type battery, thus enabling increased energy density, and replacement of organic electrolyte by an inflammable solid electrolyte can solve the safety concerns.

However, the generally poor Mg diffusivity in the solid state prevents the realisation of MIB-based devices, since divalent Mg ions strongly interact with counter anions. Therefore, it is necessary to understand the factors affecting the Mg-ion conductivity and find fast Mg-ion conductors to develop future all solid-state MIBs.

**Fig. 1:**
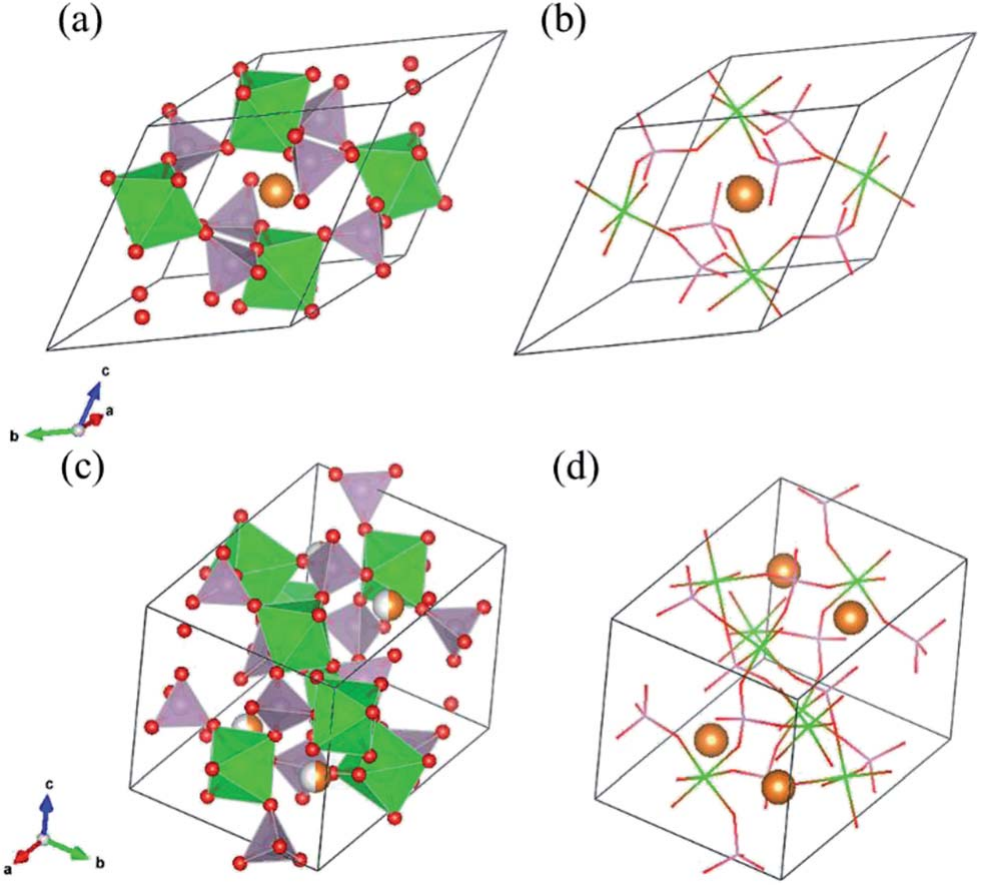
(a) and (b) show the NASICON-type structure of MZP and (c) and (d) show the β-iron sulfate-type structure of MZP with the ZrO_6_ octahedra depicted in green, PO_4_ tetrahedra depicted in lavender, O atoms in red, and Mg atoms in orange.

As solid electrolyte materials, oxide-based Na super ionic conductor (NASICON)-type structures are attractive for LIBs because NASICON-type ion conductors show high ion conductivity in both Na^3–9^ and Li^10–17^ systems. So far, one of the best Mg-ion conductors is rhombohedral NASICON-type (Mg_0.1_Hf_0.9_)_4/3.8_Nb(PO_4_)_3_ (Fig. 1(a and b)), in which the ionic conductivity of Mg^2+^ is 2.1 × 10^−6^ S cm^−1^ at 573 K and its activation energy is 0.68 eV.^18^ This compound consists of (Nb,Hf)O_6_ octahedra and PO_4_ tetrahedra alternately bridged at the two-coordinated edge O atoms, and Mg ions migrate through the interstitial sites of the corner-shared network. This local bonding rule leads to another type of structure with a different network topology, known as the monoclinic β-iron sulfate-type structure, and MgZr_4_(PO_4_)_6_ belonging to this structure type (Fig. 1(c and d)) also exhibits relatively high Mg-ion conductivity.^19,20^ An example of a material with this type of structure is Mg_0.7_(Zr_0.85_Nb_0.15_)_4_(PO_4_)_6_. The ionic conductivity of this compound is 1.1 × 10^−7^ S cm^−1^ at 573 K and the activation energy is 0.92 eV,^21^ and Mg-ion conduction of this compound at 350 °C has been recently confirmed using non-blocking Mg metal electrodes.^22^ Interestingly, both the NASICON-type and β-iron sulfate-type structural frameworks have a polymorphic relationship represented by A_*x*_B_4_(XO_4_)_6_ frameworks. In case of Na ion migration, the NASICON-type structure has been reported to show higher conductivity than that of the β-iron sulfate-type one for the composition of Na_3_Zr_2_Si_2_PO_12_.^4^ The lower activation energy for the Mg ion conduction in the NASICON-type (Mg_0.1_Hf_0.9_)_4/3.8_Nb(PO_4_)_3_ structure than in the β-iron sulfate-type Mg_0.7_(Zr_0.85_Nb_0.15_)_4_P_6_O_24_ structure has been attributed to the higher structural symmetry of the former structure.^15^ NASICON-type MgZr_4_(PO_4_)_6_ has not been synthesised primarily because the stability of the NASICON-type structure is lower for small interstitial cations like Mg^2+^ ions.^19^

However, the relationship between the structure and ionic conductivity is not yet clear for the case of Mg ions, because there are no experimental ionic conductivity data of both structures with the same composition. In this study, we evaluate the ionic conductivity for both the NASICON-type and β-iron sulfate-type structures with the stoichiometric composition of MgZr_4_(PO_4_)_6_ (MZP) using first-principles molecular dynamics (FPMD) study based on density functional theory (DFT). So far, MZP has been experimentally prepared only in the β-iron sulfate-type structure, and no report on the synthesis of the NASICON-type structure is known. We also investigated the phase stability of NASICON-type and β-iron sulfate-type structures under various temperature and pressure conditions.

## Experimental section

### Computational details

Structure relaxation parameters including the lattice parameters and internal coordinates were calculated by first-principles DFT calculations. In detail, a Vienna ab initio simulation package (VASP)^23–26^ based on DFT^27^ with a projector augmented-wave (PAW)^28,29^ method and a plane-wave basis set was used. Further, we used a generalized gradient approximation (GGA)-type exchange–correlation functional developed by Perdew, Burke, and Ernzernhof and later modified for solid materials (PBEsol).^30^ The cutoff energy for the plane-wave basis was set to 500 eV and *k*-point resolution was set to ∼1000 (*i.e.*, *N*_*x*_, *N*_*y*_, and *N*_*z*_, the number of grids in *k*_*x*_-, *k*_*y*_-, and *k*_*z*_-directions of the reciprocal space were set to satisfy *N*_*x*_ × *N*_*y*_ × *N*_*z*_ × *N*_atom_ ≈ 1000, where *N*_atom_ is the number of atoms in each unit cell).

The NASICON-type structure was constructed by replacing Li ions in the structure of LiZr_2_(PO_4_)_3_ taken from the inorganic crystal structure database (ICSD) (ID: 92250, original data reported by Catti *et al.*)^31^ with Mg ions and vacancies, because there are no reports of the experimental synthesis. The crystal structure input for β-iron sulfate-type MZP was extracted from the ICSD (ID: 250452, original data reported by Gobechiya *et al.*).^32^

Table 1 lists DFT-derived relaxed lattice parameters, cell volume, and calculated total electron energies for (a) NASICON-type and (b) β-iron sulfate-type MZP. In the NASICON-type structure, three Mg ions and vacancies reside at the 6b and/or 36f Wyckoff position (36f sites are split around the 6b site). The most stable Mg/vacancy arrangement was determined using a genetic algorithm (GA), as described in our previous paper.^33–35^ Mg-ion occupation at 6b sites is the most stable after GA optimisation, and the energy difference among the GA-derived structures (2400 samples) is within 60 meV per atom.

**Table 1:** DFT-derived relaxed lattice parameters, cell volume, and calculated total electron energies for the most stable structure of (a) NASICON-type and (b) β-iron sulfate-type MZP

	*a*/Å	*b*/Å	*c*/Å	*α*/°	*β*/°	*γ*/°	Cell volume per atom/Å^3^ per atom	Electron energy per atom/eV per atom
(a)	8.91	8.98	22.4	89.80	89.99	119.99	14.8	−8.041
(b)	12.52	8.97	8.95	90.00	90.49	90.00	14.4	−8.055

In contrast, Mg ions and vacancies occupied the 4e sites of the Wyckoff position at 1 : 1 molar ratio in the β-iron sulfate-type MZP. Three configurations of Mg/vacancy arrangements were calculated for the unit cell of Mg_2_Zr_8_P_12_O_48_, and the most stable one was chosen for following calculations. The volumetric and energetic differences among the three configurations are 0.19 Å^3^ per atom and 12 meV per atom, respectively, indicating a small dependence on the Mg arrangement.

The obtained Mg/vacancy configurations for NASICON-type and β-iron sulfate-type MZP structures and their lattice parameters were thereafter used as structural inputs for following calculations, unless specified otherwise.

As listed in Table 1, the β-iron sulfate-type MZP is slightly more stable than the NASICON-type one by only 14 meV per atom, and the cell volume per atom difference between β-iron sulfate-type MZP (14.4 Å^3^ per atom) and NASICON-type (14.8 Å^3^ per atom) is small. Therefore, both structures are reasonably stable and are expected to be good candidates for comparing the Mg-ion diffusivity.

FPMD calculations were performed to compare the Mg-ion conductivity of the NASICON-type and the β-iron sulfate-type MZP structures. Initially, all the Mg atoms were located in the 6b sites for the NASICON-type structure and 4e sites for the β-iron sulfate-type structure.

The supercell structure including eight MZP units (*i.e.*, Mg_8_Zr_32_P_48_O_192_) was prepared to evaluate the Mg-ion conductivity *via* FPMD simulations. The lattice constants of the supercell of the NASICON-type and β-iron sulfate-type MZP are *a* = 12.54 Å, *b* = 17.62 Å, *c* = 17.64 Å, *α* = 89.99°, *β* = 90.01°, *γ* = 89.00° and *a* = 12.52 Å, *b* = 17.94 Å, *c* = 17.90 Å, *α* = 90.00°, *β* = 90.49°, and *γ* = 90.00°, respectively. The lattice vectors **a**_sc_, **b**_sc_, and **c**_sc_ in the supercell are described by a linear combination of lattice vectors **a**_p_, **b**_p_, and **c**_p_ in a primitive cell of the NASICON-type or β-iron sulfate-type structure, as shown below:(**a**_sc_**b**_sc_**c**_sc_) = (**a**_p_**b**_p_**c**_p_)**M**_sc_where, the supercell matrix **M**_sc_ is expressed as follows:
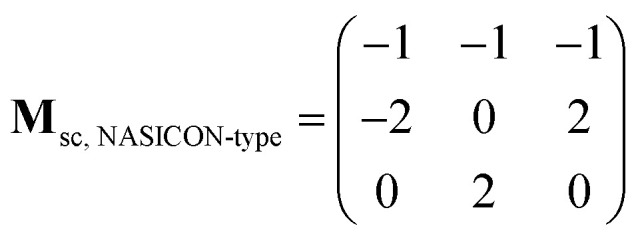

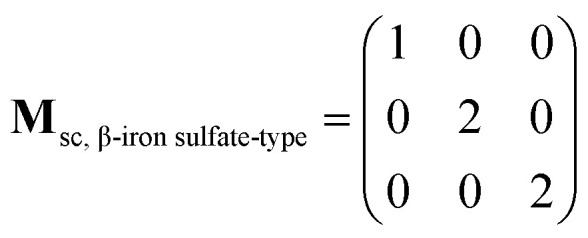


FPMD simulations were performed for NASICON-type and β-iron sulfate-type structure in the temperature range of 1573 to 1973 K with increments of 50 K for 100 ps. The DFT calculation settings used were equivalent to those of the structure relaxation calculations except for the cutoff energy and *k*-point grids as follows: the cutoff energy for the plane-wave basis was set to 350 eV and 1 × 1 × 1 *k*-point grid (only Γ point) was employed to reduce the computational cost. For MD simulations, the time step was set to 1 fs, and the NVT canonical ensemble was adopted using a Nosé thermostat.^36^ The mean square displacements (MSD) of all elements were calculated, and then the diffusion coefficients of Mg-ion conduction and corresponding migration energies were determined. Above FPMD studies have been performed in our previous studies, showing reasonable accordance between the experimental and the computational results.^10,11,37^

We evaluated the thermodynamic stability of the MZP polymorphs by first principles DFT calculations. Temperature- and/or pressure-dependent phase stability was also evaluated using DFT-based phonon calculations or cell-volume constraint calculations, respectively. We used the same lattice as that used for FPMD simulations. Structural relaxation calculations were performed for a structure in which the lattice constant was varied from 95 to 103% under the condition of constant lattice volume. The energy–volume relationship is fitted with the Murnaghan equation of state:^38,39^1
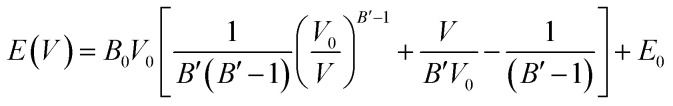
where, *B*_0_ is the bulk modulus at zero pressure, *B*′ is its first derivative, *E*_0_ is the minimum energy, and *V*_0_ is the volume at the minimum energy. In addition, the bulk modulus *B* can be expressed as,

2 *B* = −*V*d*p*/d*V*

where, *V* and *p* are the volume and pressure, respectively. The enthalpy–pressure diagram was constructed by combining eqn (1) and (2).

Phonon frequencies and their contribution to free energy changes were calculated using the PHONOPY code.^40^ Density functional perturbation theory (DFPT),^41,42^ also implemented in VASP, was used to estimate the real-space force constants of the supercells. The other calculation conditions were the same as those used for the structure relaxation calculations.

From these calculations, the free energy was calculated by changing the temperature from 0 to 2000 K and the pressure was changed from 0 to 20 GPa.

## Results and discussion

**Fig. 2:**
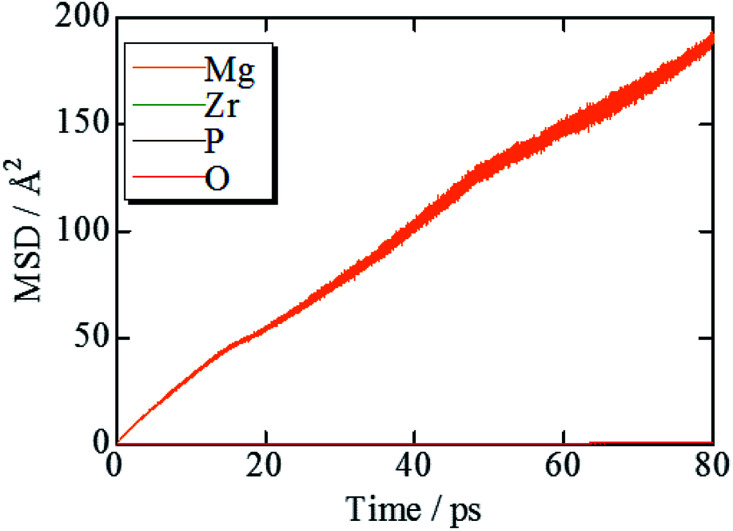
MSD plots of Mg, Zr, P, and O trajectories in β-iron sulfate-type MZP at 1973 K.

Fig. 2 shows the evolution of the time-averaged mean square displacement (MSD) for constituent ions in MZP with β-iron sulfate-type structure. The MSD of Mg ions increases linearly with the MD step (time), indicating that Mg^2+^ ions diffuse over the lattice. In contrast, the MSD profiles of the other ions remains constant (<1.1 Å^2^). Therefore, Zr, P, and O ions remain near their original sites during the MD run, and these MSD values represent the magnitude of thermal vibrations. Similar MSD profiles were obtained for the NASICON-type structure, in which only Mg ions are found to diffuse in the lattice.

**Fig. 3:**
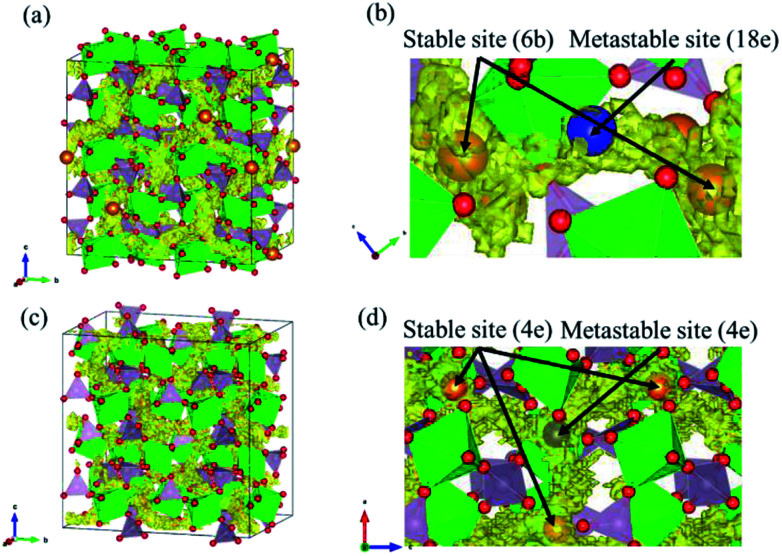
Trace of Mg atom in FPMD simulation at 1973 K. (a), (b) and (c), (d) show the NASICON-type and β-iron sulfate-type structures, respectively. In (b) and (d), the stable and metastable sites of Mg are denoted in orange and blue, respectively.

Diffusion path of Mg ions at 1573 K and 1973 K, *i.e.* both the lowest and the highest simulation temperatures, are visualised through isosurfaces of the Mg-ion probability density distribution from the FPMD simulation for (Fig. 3(a and b) and ESI Fig. S1(a)†) the NASICON-type and (Fig. 3(c and d) and ESI Fig. S1(b)†) β-iron sulfate-type structures. We confirmed the formation of a 3-dimensional pathway for both structures. Moreover, metastable Mg-ion sites are indicated in both (Fig. 3b) NASICON-type and (Fig. 3d) β-iron sulfate-type structures by varying the isosurface level (Li ion probability density). In Fig. 3b and d, the stable and metastable sites are marked with orange and blue circles, respectively. On one hand, the metastable Mg sites in β-iron sulfate-type structure are linked to three neighbouring 4e stable Mg/vacancy sites with a straight diffusion path. On the other hand, the metastable Mg sites (18e sites) are connected to two neighbouring 6b stable sites, which is consistent with the reported Li migration pathway in LiZr_2_(PO_4_)_3_ and its derivative.^43^

Diffusion coefficients were extracted through a simple linear fitting with the following expression:

3 〈*x*^2^〉 = 2*nDt*

where, *n*, *D*, and *t* represent the dimension of Mg-ion diffusion, ionic diffusion coefficient, and time, respectively. The ionic conductivity of Mg is estimated by the Nernst–Einstein relationship:4
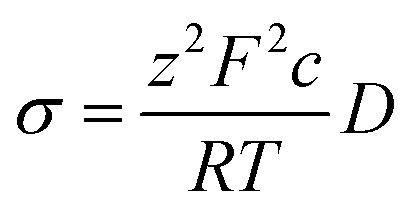
where, *R*, *T*, *z*, *F*, and *c* represent the gas constant, temperature, ionic valence, Faraday constant, and ionic concentration, respectively. Since number of Mg^2+^ hopping events are limited within the simulation time (100 ps) at ambient temperature according to Boltzmann statistics and attempt frequency, high temperature simulation (>1573 K) is necessary to evaluate migration energy in sufficient accuracy. Therefore, low temperature conductivities are extrapolated from high temperature data as reported in our previous FPMD calculations.^10,11,37^

**Fig. 4:**
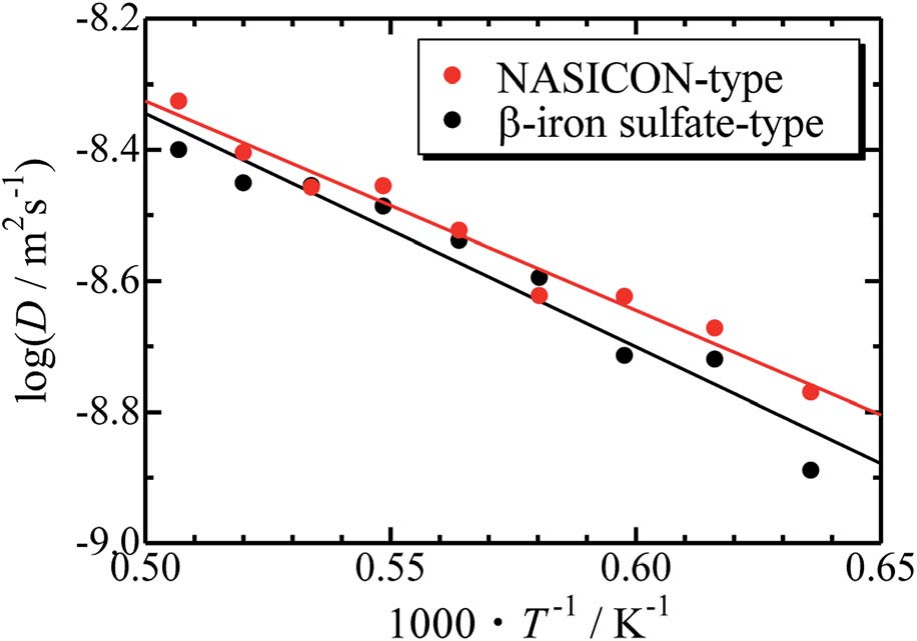
Arrhenius plots of the temperature-dependent Mg-ion diffusion coefficients fitted with a straight line.

The Arrhenius plots for the NASICON-type and the β-iron sulfate-type structures is shown in Fig. 4, and the migration energies of Mg were evaluated by a simple linear fitting scheme. Higher Mg-ion conductivity and lower migration energy are derived for the NASICON-type structure compared to those of the β-iron sulfate-type structure.

The migration energy of Mg in the β-iron sulfate-type structure obtained in this study (0.71 eV) is different from the experimental value (1.6 eV),^21^ which may owe to inclusion of grain boundary conduction for experimental results. The β-iron sulfate-type structure has a higher migration energy than the NASICON-type structure (0.63 eV) in present computations. This tendency agrees that the migration energy of β-iron sulfate-type Mg_0.7_(Zr_0.85_Nb_0.15_)_4_(PO_4_)_6_ (0.92 eV)^21^ is higher than that of NASICON-type (Mg_0.1_Hf_0.9_)_4/3.8_Nb(PO_4_)_3_ (0.68 eV).^18^ This result suggests that the NASICON-type structure has better ion conductivity than the β-iron sulfate-type one.

**Fig. 5:**
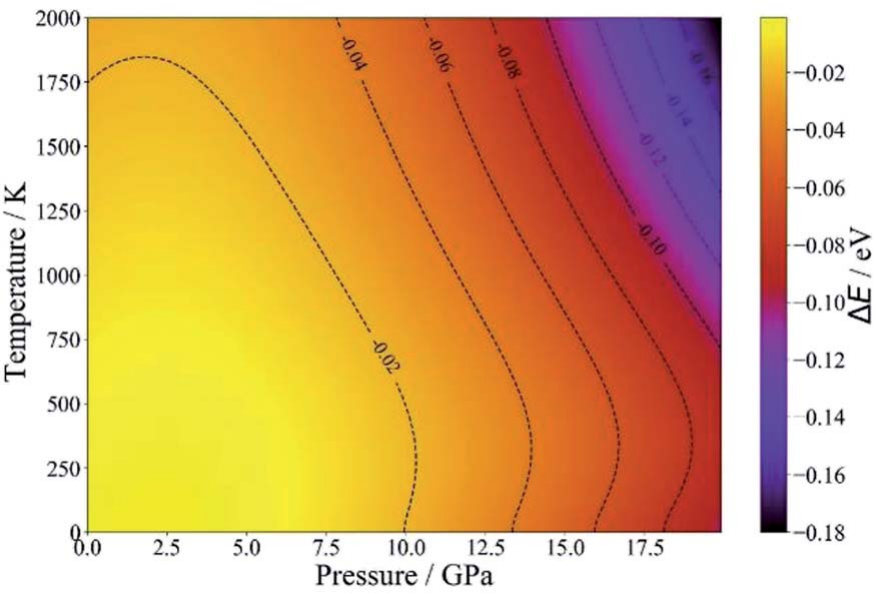
Temperature- and pressure-dependent free energies of β-iron sulfate-type and NASICON-type MZP.

Since NASICON-type MZP has higher Mg-ion conductivity, the pressure- and temperature-dependence of the phase stability was investigated for NASICON-type and β-iron sulfate-type structures. Fig. 5 displays the difference between the phase stability of β-iron sulfate-type structure and NASICON-type one, Δ*E*, as functions of temperature and pressure. Δ*E* is described as follows:

5 Δ*E* = *E*(*β*-iron sulfate-type) − *E*(NASICON-type)

where, *E*(β-iron sulfate-type) and *E*(NASICON-type) represent the free energies of the corresponding structures at a given pressure and temperature. Since Δ*E* is negative for all pressure and temperature conditions, the formation of the NASICON-type structure is thermodynamically disfavoured. However, the energy difference is only 14 meV per atom between two structures (at 0 K and at 0 atm), so that formation of NASICON-type structure may be feasible such as by doping techniques. Also, since the NASICON-type structure is stable under negative pressure condition, thin film formation technique using selection of appropriate substrate may stabilizes NASICON-type structure. In addition, we computed phase stability of MgTi_4_(PO_4_)_6_ composition by DFT. NASICON-type structure is more stable than β-iron sulfate-type structure by 7 meV per atom, showing accordance with experimental formation of NASICON-type structure.^44^ These results validate the present computational results.

## Conclusions

First-principles DFT calculations were performed to clarify the difference between the Mg-ion conductivity in β-iron sulfate-type and NASICON-type MZP. The results of the FPMD calculations suggest that the NASICON-type structure has higher Mg-ion conductivity than the β-iron sulfate-type one. However, evaluation of the pressure- and temperature-dependent free energies by DFT calculations indicated that the formation of NASICON-type MZP is disfavoured. Therefore, the composition should be appropriately optimised to stabilize the NASICON-type structure.

## Conflicts of interest

There are no conflicts to declare.

## Supplementary Material

RA-009-C9RA00513G-s001

## References

[cit1] Van Noorden R. (2014). Nature.

[cit2] Srour H., Chancelier L., Bolimowska E., Gutel T., Mailley S., Rouault H., Santini C. C. (2016). J. Appl. Electrochem..

[cit3] Deng Y., Eames C., Nguyen L. H. B., Pecher O., Griffith K. J., Courty M., Fleutot B., Chotard J.-N., Grey C. P., Islam M. S., Masquelier C. (2018). Chem. Mater..

[cit4] Goodenough J. B., Hong H.-P., Kafalas J. A. (1976). Mater. Res. Bull..

[cit5] Hong H.-P. (1976). Mater. Res. Bull..

[cit6] Zhou W., Li Y., Xin S., Goodenough J. B. (2017). ACS Cent. Sci..

[cit7] Lalère F., Leriche J. B., Courty M., Boulineau S., Viallet V., Masquelier C., Seznec V. (2014). J. Power Sources.

[cit8] Noguchi Y., Kobayashi E., Plashnitsa L. S., Okada S., Yamaki J. (2013). Electrochim. Acta.

[cit9] Jolley A. G., Cohn G., Hitz G. T., Wachsman E. D. (2015). Ionics.

[cit10] Noda Y., Nakano K., Takeda H., Kotobuki M., Lu L., Nakayama M. (2017). Chem. Mater..

[cit11] Noda Y., Nakano K., Otake M., Kobayashi R., Kotobuki M., Lu L., Nakayama M. (2018). APL Mater..

[cit12] Zhao X., Zhang Z., Zhang X., Tang B., Xie Z., Zhou Z. (2018). J. Mater. Chem. A.

[cit13] Knauth P. (2009). Solid State Ionics.

[cit14] Thangadurai V., Weppner W. (2006). Ionics.

[cit15] Bachman J. C., Muy S., Grimaud A., Chang H.-H., Pour N., Lux S. F., Paschos O., Maglia F., Lupart S., Lamp P., Giordano L., Shao-Horn Y. (2016). Chem. Rev..

[cit16] Yao X., Huang B., Yin J., Peng G., Huang Z., Gao C., Liu D., Xu X. (2016). Chin. Phys. B.

[cit17] Cao C., Li Z.-B., Wang X.-L., Zhao X.-B., Han W.-Q. (2014). Front. Energy Res..

[cit18] Tamura S., Yamane M., Hoshino Y., Imanaka N. (2016). J. Solid State Chem..

[cit19] Nomura K., Ikeda S., Ito K., Einaga H. (1992). Bull. Chem. Soc. Jpn..

[cit20] Ikeda S., Takahashi M., Ishikawa J., Ito K. (1987). Solid State Ionics.

[cit21] Imanaka N., Okazaki Y., Adachi G. (1999). Electrochem. Solid-State Lett..

[cit22] Kajihara K., Nagano H., Tsujita T., Munakata H., Kanamura K. (2017). J. Electrochem. Soc..

[cit23] Kresse G., Hafner J. (1993). Phys. Rev. B: Condens. Matter Mater. Phys..

[cit24] Kresse G., Furthmüller J. (1996). Phys. Rev. B: Condens. Matter Mater. Phys..

[cit25] Kresse G., Furthmüller J. (1996). Comput. Mater. Sci..

[cit26] Kresse G., Hafner J. (1994). Phys. Rev. B: Condens. Matter Mater. Phys..

[cit27] Hohenberg P., Kohn W. (1964). Phys. Rev..

[cit28] Kresse G., Joubert D. (1999). Phys. Rev. B: Condens. Matter Mater. Phys..

[cit29] Blöchl P. E. (1994). Phys. Rev. B: Condens. Matter Mater. Phys..

[cit30] Perdew J. P., Ruzsinszky A., Csonka G. I., Vydrov O. A., Scuseria G. E., Constantin L. A., Zhou X., Burke K. (2008). Phys. Rev. Lett..

[cit31] Catti M., Stramare S. (2000). Solid State Ionics.

[cit32] Gobechiya E. R., Sukhanov M. V., Pet'kov V. I., Kabalov Y. K. (2008). Crystallogr. Rep..

[cit33] Trimarchi G., Zunger A. (2007). Phys. Rev. B: Condens. Matter Mater. Phys..

[cit34] Abraham N. L., Probert M. I. J. (2006). Phys. Rev. B: Condens. Matter Mater. Phys..

[cit35] Woodley S. M., Battle P. D., Gale J. D., Catlow C. R. A. (1999). Phys. Chem. Chem. Phys..

[cit36] Nosé S. (1984). J. Chem. Phys..

[cit37] Jalem R., Yamamoto Y., Shiiba H., Nakayama M., Munakata H., Kasuga T., Kanamura K. (2013). Chem. Mater..

[cit38] Birch F. (1947). Phys. Rev..

[cit39] Birch F. (1938). J. Appl. Phys..

[cit40] Togo , Phonopy, https://atztogo.github.io/phonopy/, (accessed 5 December 2018)

[cit41] Refson K., Tulip P. R., Clark S. J. (2006). Phys. Rev. B: Condens. Matter Mater. Phys..

[cit42] Baroni S., de Gironcoli S., Dal Corso A., Giannozzi P. (2001). Rev. Mod. Phys..

[cit43] Padma kumar P., Yashonath S. (2001). J. Phys. Chem. B.

[cit44] Takahashi H., Takamura H. (2012). Mater. Trans..

[cit45] Momma K., Izumi F. (2011). J. Appl. Crystallogr..

