# Supplementary material for: Computational investigation of the Mg-ion conductivity and phase stability of MgZr4(PO4)6
Source: RSC Adv. 2019 Apr 23;9(22):12590–5. doi: 10.1039/c9ra00513g (PMC9063713; doi:10.1039/c9ra00513g)
Supplement: RA-009-C9RA00513G-s001 [file RA-009-C9RA00513G-s001.pdf]

Electronic Supplementary Material (ESI) for RSC Advances.
This journal is © The Royal Society of Chemistry 2019

Electronic Supplementary Information

Computational investigation of the Mg-ion conductivity and phase stability of $MgZr_4(PO_4)_6$

Koki Nakano,[a] Yusuke Noda,[b] Naoto Tanibata,[a,c] Masanobu Nakayama,*[a,b,c,d,e] Koichi Kajihara,[f] Kiyoshi Kanamura[f]

a. Department of Advanced Ceramics, Nagoya Institute of Technology, Gokiso, Showa, Nagoya, Aichi 466-8555, Japan

b. Center for Materials research by Information Integration (CMI2), Research and Services Division of Materials Data and Integrated System (MaDIS), National Institute for Materials Science (NIMS), 1-2-1 Sengen, Tsukuba, Ibaraki 305-0047, Japan

c. Elements Strategy Initiative for Catalysts and Batteries (ESICB), Kyoto University, 1-30 Goryo-Ohara, Nishikyo, Kyoto 615-8245, Japan

d. Frontier Research Institute for Materials Science (FRIMS), Nagoya Institute of Technology, Gokiso, Showa, Nagoya, Aichi 466-8555, Japan

e. Global Research Center for Environment and Energy based on Nanomaterials Science (GREEN), National Institute for Materials Science (NIMS), 1-1 Namiki, Tsukuba, Ibaraki 305-0047, Japan

f. Department of Applied Chemistry for Environment,Graduate School of Urban Environmental Sciences, Tokyo Metropolitan University,1-1 Minami-Osawa, Hachioji, Tokyo 192-0397, Japan

(a) (b)

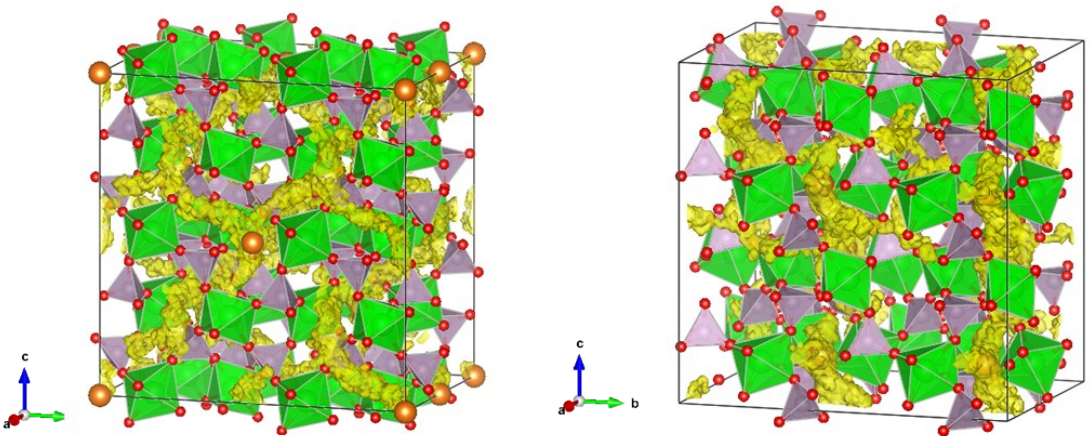

Supporting Figure S1. Trace of Mg atom in FPMD simulation at 1573 K. (a) and (b) show the NASICON-type and β−iron sulfate-type structures, respectively.

Supporting Table S1. Mg-Ion Conductivity (σ_{Mg}) and Activation Energy (E_{act}) of Mg-Ion Conductors

Study	σ_{Mg} [S cm^{-1}]	E_{act} [eV]
Theoretical (β-iron sulfate-type, this paper)	3.9×10^{-3} (bulk, 873 K)	0.71
Theoretical (NASICON-type, this paper)	7.1×10^{-3} (bulk, 873 K)	0.63
Experimental[21] (β-iron sulfate-type)	1×10^{-5} (873 K)	1.6
Experimental[21] $Mg_{0.7}(Zr_{0.85}Nb_{0.15})_4P_6O_{24}$ (β-iron sulfate-type)	1.1×10^{-7} (573 K)	0.92
Experimental[22] $Mg_{0.7}(Zr_{0.85}Nb_{0.15})_4P_6O_{24}$ (β-iron sulfate-type)	1.1×10^{-6} (total, 623 K)	1.18
Experimental[18] $(Mg_{0.1}Hf_{0.9})_{4/3.8}Nb(PO_4)_3$ (NASICON-type)	2.1×10^{-6} (bulk, 573 K)	0.68
